# Supplementary material for: Metabolic and Antioxidant Variations in “Regina” Raspberries: A Comparative Analysis of Early and Late Harvests
Source: Plants (Basel). 2025 Mar 12;14(6):888. doi: 10.3390/plants14060888 (PMC11946257; doi:10.3390/plants14060888)
Supplement: Supplementary file 1 [file plants-14-00888-s001.zip › Supplementary Table S2.pdf]

Supplementary Table S2. Significant features identified by Volcano Plot Analysis (Cut-off: Fold Change >1 and Adjusted p-value <0.05)

Compounds	FC	log2(FC)	p.ajusted	-LOG10(pvalue)
Apigenin 6,8-C-arabinoside-C-glucoside	0,20796	-2,2656	2,77E-05	4,5575
Apigenin 6,8-C-galactoside-C-arabinoside	0,20811	-2,2646	2,77E-05	4,5575
Apigenin 7-O-apiosyl-glucoside	0,21019	-2,2502	2,77E-05	4,5575
Orobol	0,37533	-1,4138	2,77E-05	4,5575
5,6,7,4'-Tetrahydroxyisoflavone	0,37585	-1,4118	2,77E-05	4,5575
Luteolin	0,37593	-1,4115	2,77E-05	4,5575
7,8,3',4'-Tetrahydroxyisoflavone	0,37625	-1,4102	2,77E-05	4,5575
Scutellarein	0,38253	-1,3863	2,77E-05	4,5575
Cyanidin 3-(2G-xylosylrutinoside)	0,2215	-2,1746	6,63E-05	4,1784
4-Hydroxybenzoic acid 4-O-glucoside	3,9371	1,9771	7,10E-05	4,1485
Cyanidin 3-O-glucosyl-rutinoside	0,30649	-1,7061	7,10E-05	4,1485
Peonidin 3-sambubioside 5-glucoside	0,30649	-1,7061	7,10E-05	4,1485
8-Hydroxygenistein	0,34418	-1,5388	7,10E-05	4,1485
Kaempferol	0,34592	-1,5315	7,10E-05	4,1485
6,7,3',4'-Tetrahydroxyisoflavone	0,3471	-1,5266	7,10E-05	4,1485
Dihydrodaidzein 7-glucuronide	0,42483	-1,235	7,10E-05	4,1485
Apigenin 6-C-glucoside	0,43078	-1,215	7,10E-05	4,1485
Genistin	0,44291	-1,1749	7,10E-05	4,1485
3'-Hydroxygenistein	0,35121	-1,5096	7,26E-05	4,139
Kaempferol 3-O-glucosyl-rhamnosyl-galactoside	0,30728	-1,7024	7,30E-05	4,1367
Kaempferol 3-O-glucosyl-rhamnosyl-glucoside	0,30728	-1,7024	7,30E-05	4,1367
Petunidin 3-O-(6''-p-coumaroyl-glucoside)	0,37773	-1,4046	7,30E-05	4,1367
Pinotin A	0,37773	-1,4046	7,30E-05	4,1367
Procyanidin trimer EEC	0,22769	-2,1349	8,36E-05	4,078
Procyanidin trimer C1	0,22815	-2,1319	8,36E-05	4,078
Procyanidin trimer C2	0,23965	-2,061	8,36E-05	4,078
Procyanidin trimer T2	0,24342	-2,0385	8,36E-05	4,078
Pelargonidin 3-glucoside	0,3062	-1,7075	8,36E-05	4,078
Pelargonidin 3-O-galactoside	0,3062	-1,7075	8,36E-05	4,078
3-(3,4-Dihydroxyphenyl) lactic acid	0,31283	-1,6766	8,36E-05	4,078
Syringic acid	0,31295	-1,676	8,36E-05	4,078
Cyanidin 3-rutinoside	0,31483	-1,6674	8,36E-05	4,078
Petunidin 3-O-rutinoside	0,31483	-1,6674	8,36E-05	4,078
Pelargonidin 3-(2gluglucosylrutinoside)	0,32821	-1,6073	8,36E-05	4,078
Eriocitrin	0,33068	-1,5965	8,36E-05	4,078
Neoeriocitrin	0,33463	-1,5793	8,36E-05	4,078
Kaempferol 3-O-xylosyl-rutinoside	0,34157	-1,5498	8,36E-05	4,078
Kaempferol 3-O-rhamnosyl-rhamnosyl-glucoside	0,34392	-1,5399	8,36E-05	4,078
Luteolin 7-O-glucoside	0,36675	-1,4471	8,36E-05	4,078
Kaempferol 3-O-galactoside	0,36675	-1,4471	8,36E-05	4,078
Kaempferol 3-O-glucoside	0,36675	-1,4471	8,36E-05	4,078
Dihydroquercetin 3-O-rhamnoside	0,36952	-1,4363	8,36E-05	4,078

Eriodictyol 7-O-glucoside	0,37177	-1,4275	8,36E-05	4,078
Phloretin 2'-O-glucuronide	0,37255	-1,4245	8,36E-05	4,078
4-p-Coumaroylquinic acid	0,4826	-1,0511	8,36E-05	4,078
Dihydrosinapic acid	0,31811	-1,6524	9,41E-05	4,0265
Cyanidin 3-galactoside	0,37668	-1,4086	9,41E-05	4,0265
Cyanidin 3-glucoside	0,37668	-1,4086	9,41E-05	4,0265
Dihydroferuloylglycine	0,38518	-1,3764	9,98E-05	4,0007
Tectorigenin	0,40688	-1,2973	9,98E-05	4,0007
Neodiosmin	0,31618	-1,6612	0,00010112	3,9952
Peonidin 3-sambubioside	0,33496	-1,5779	0,00010764	3,968
Isopeonidin 3-sambubioside	0,33503	-1,5776	0,00010764	3,968
Narirutin 4'-O-glucoside	0,35515	-1,4935	0,00010764	3,968
Isotectorigenin	0,41616	-1,2648	0,00010764	3,968
3'-Hydroxymelanettin	0,41743	-1,2604	0,00010764	3,968
Luteolin 6-C-glucoside	0,37343	-1,4211	0,00010949	3,9606
Ethyl gallate	0,32403	-1,6258	0,00011956	3,9224
3,4-O-Dimethylgallic acid	0,32603	-1,6169	0,00011956	3,9224
Episesaminol	0,34458	-1,5371	0,00013771	3,861
Sesaminol	0,34458	-1,5371	0,00013771	3,861
Sesamolin	0,34458	-1,5371	0,00013771	3,861
Dihydroferulic acid 4-glucuronide	3,3151	1,7291	0,00015562	3,8079
Peonidin 3-sophoroside	0,44994	-1,1522	0,00016513	3,7822
1,2,3-Benzenetriol	0,43576	-1,1984	0,00016948	3,7709
Kaempferol 3-O-galactoside 7-O-rhamnoside	0,30049	-1,7346	0,00017695	3,7522
Procyanidin B1	0,42238	-1,2434	0,0001887	3,7242
Myricetin 3-O-rutinoside	3,6636	1,8733	0,00019148	3,7179
Quercetin 3,4'-diglucoside	3,6636	1,8733	0,00019148	3,7179
Quercetin 3-O-sophoroside	3,6636	1,8733	0,00019148	3,7179
Quercetin 7,4'-O-diglucoside	3,6636	1,8733	0,00019148	3,7179
Kaempferol 3-O-rutinoside	0,32975	-1,6005	0,00019148	3,7179
Luteolin 7-O-rutinoside	0,33194	-1,591	0,00019148	3,7179
Quercetin 3-O-glucosyl-rhamnosyl-galactoside	2,6847	1,4247	0,00019148	3,7179
Quercetin 3-O-glucosyl-rhamnosyl-glucoside	2,6847	1,4247	0,00019148	3,7179
Isorhamnetin 3-O-glucoside 7-O-rhamnoside	0,49032	-1,0282	0,00019148	3,7179
Apigenin 6,8-di-C-glucoside	0,3333	-1,5851	0,00019354	3,7132
Quercetin 4'-glucuronide	0,12923	-2,952	0,00019466	3,7107
Quercetin 3-O-galactoside 7-O-rhamnoside	2,8984	1,5353	0,00019466	3,7107
Quercetin 3-O-rhamnosyl-galactoside	2,8984	1,5353	0,00019466	3,7107
Quercetin 3-rutinoside	2,8984	1,5353	0,00019466	3,7107
4'-Methyldelphinidin 3-rutinoside	0,47604	-1,0709	0,00019466	3,7107
Cyanidin 3-sambubioside	0,48569	-1,0419	0,00019972	3,6996
Koparin	0,39556	-1,338	0,0002025	3,6936
Gallic acid	0,4221	-1,2444	0,00022602	3,6459
(+)-Catechin 3-O-glucose	0,45954	-1,1217	0,00022602	3,6459
Ligstroside	3,8501	1,9449	0,00023688	3,6255
Petunidin 3-arabinoside	0,37317	-1,4221	0,00028418	3,5464

Naringin 4'-O-glucoside	0,28451	-1,8135	0,00030803	3,5114
Peonidin 3-arabinoside	0,37013	-1,4339	0,00031296	3,5045
Peonidin 3-xyloside	0,37416	-1,4183	0,00031296	3,5045
Catechin	2,9435	1,5575	0,00031384	3,5033
Epicatechin	2,9259	1,5489	0,00032557	3,4874
Quercetin 3'-glucuronide	0,149	-2,7466	0,00038846	3,4106
Hydroxytyrosol	3,027	1,5979	0,00048052	3,3183
Luteolin 7-O-(2-apiosyl-glucoside)	0,48829	-1,0342	0,00048795	3,3116
Isopropyl 3-(3,4-dihydroxyphenyl)-2-hydroxypropanoate	3,0926	1,6288	0,0004933	3,3069
Chrysoeriol 7-O-(6"-malonyl-apiosyl-glucoside)	2,4395	1,2866	0,00053448	3,2721
Isopeonidin 3-arabinoside	0,33422	-1,5811	0,00056965	3,2444
Isopeonidin 3-xyloside	0,33422	-1,5811	0,00056965	3,2444
Cyanidin 3-diglucoside 5-glucoside	0,45125	-1,148	0,00059019	3,229
Kaempferol 3-O-xylosyl-glucoside	0,49598	-1,0117	0,00059163	3,2279
Cyanidin 3-xyloside	3,0524	1,61	0,00059504	3,2255
Kaempferol 3-O-sophoroside 7-O-glucoside	0,43447	-1,2027	0,0006236	3,2051
Kaempferol 3,7,4'-O-triglucoside	0,43712	-1,1939	0,00063602	3,1965
Esculin	2,4914	1,317	0,00064159	3,1927
Cyanidin 3-arabinoside	2,983	1,5768	0,00072286	3,1409
Kaempferol 3-O-(2"-rhamnosyl-galactoside) 7-O-rhamnoside	0,38038	-1,3945	0,00072734	3,1383
Hispidulin	2,2402	1,1636	0,00074855	3,1258
Phenylacetylglycine	0,49525	-1,0138	0,00074855	3,1258
Poncirin	5,1392	2,3615	0,0010305	2,9869
Isorhamnetin 3-glucuronide	3,5925	1,845	0,0011595	2,9357
Isorhamnetin 4'-glucuronide	3,5925	1,845	0,0011595	2,9357
Isoquercitrin	0,48755	-1,0364	0,0013755	2,8615
Delphinidin 3-glucoside	0,38323	-1,3837	0,0015854	2,7999
Cyanidin 3-O-(6"-malonyl-3"-glucosyl-glucoside)	4,8851	2,2884	0,0031815	2,4974
Myricetin 3-O-rhamnoside	0,47148	-1,0847	0,0031815	2,4974
Quercetin 4'-O-glucoside	0,47631	-1,07	0,0033929	2,4694
Myricetin 3-O-arabinoside	0,40655	-1,2985	0,0034357	2,464
2-Hydroxyenterodiol	0,41806	-1,2582	0,0063437	2,1977
6-Hydroxydihydrodaidzein	7,6314	2,9319	0,022427	1,6492
8-Hydroxydihydrodaidzein	7,6314	2,9319	0,022427	1,6492
Syringaldehyde	7,1283	2,8335	0,02262	1,6455
3-(3,4-Dihydroxyphenyl) propanoic acid	7,1283	2,8335	0,02262	1,6455
5-p-Coumaroylquinic acid	6,5059	2,7017	0,024199	1,6162
3,4-Dihydroxy-5-methoxybenzoic acid	5,5293	2,4671	0,030279	1,5189
3,5-Dihydroxy-4-methoxybenzoic acid	5,5293	2,4671	0,030279	1,5189
Delphinidin 3-O-feruloyl-glucoside	6,3162	2,6591	0,033717	1,4722
Caffeoyl C1-glucuronide	4,4236	2,1452	0,049281	1,3073
